# Correction: Natural bamboo leaves as dielectric layers for flexible capacitive pressure sensors with adjustable sensitivity and a broad detection range

**DOI:** 10.1039/d1ra90133h

**Published:** 2021-07-27

**Authors:** Zhihao Liu, Tianlong Liang, Yue Xin, Jinhao Huang, Jionghong Liang, Xiang He, Chi Zhang, Weijia Yang, Xin He

**Affiliations:** School of Applied Physics and Materials, Wuyi University Jiangmen 529020 Guangdong P. R. China xin3231946@163.com hexinwyu@126.com

## Abstract

Correction for ‘Natural bamboo leaves as dielectric layers for flexible capacitive pressure sensors with adjustable sensitivity and a broad detection range’ by Zhihao Liu *et al.*, *RSC Adv.*, 2021, **11**, 17291–17300. DOI: 10.1039/D1RA03207K.

The authors regret that an incorrect image was used for Fig. 3e in the original article. The correct figure is provided here.

**Fig. 3:**
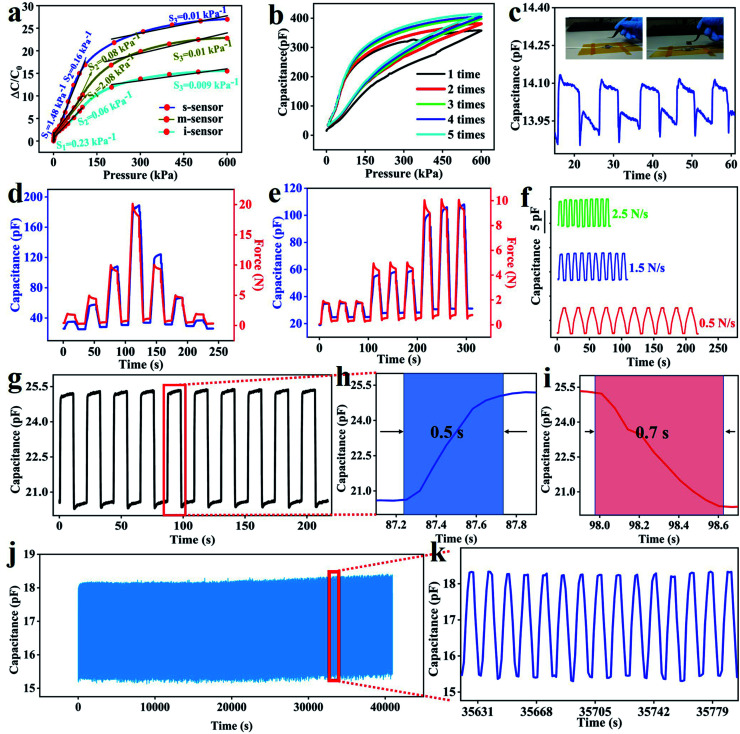


The Royal Society of Chemistry apologises for these errors and any consequent inconvenience to authors and readers.

## Supplementary Material

